# Reply to: Mucosal histopathology in celiac disease: a rebuttal of Oberhuber´s subdivision of Marsh III

**Published:** 2015

**Authors:** Georg Oberhuber, Harald Vogelsang

**Affiliations:** *Department of Pathology, Überlingen and Department of Hepatology and Gastroenterology, Internal Medicine III, University of Vienna, Austria *

**To The Editor:**

We read with interest the paper entitled “Mucosal histopathology in celiac disease: a rebuttal of Oberhuber´s sub-division of Marsh III” by MN Marsh et al. published in this Journal (1).

We have proposed a standardized classification system of the celiac lesions in 1999 (2).In their paper Marsh et al. comment on this work. To our surprise, the authors of the paper suggest that the flat mucosa (type IIIc in the modified Marsh classification) is the only acceptable and a provable stage of the destructive type of a CD lesion. According to their opinion, stages IIIa and b do not exist. They support their notion by comparing the scanning electron microscopy (SEM) of two IIIc lesions in fig. 2 and 3 accompanied by the corresponding conventional histological picture. In fig. 3 they demonstrate a section that could be mistaken for a “villous” projection as depicted in their figure legend. In our opinion only an inexperienced histopathologist would classify this lesion as IIIb. In fig. 6 they furthermore demonstrate that the severity of mucosal atrophy could be underestimated in histological sections passing through a collared basin. Unfortunately they only presented a cartoon to elucidate this type of error. We doubt that they are able to demonstrate such a lesion on histological sections. Furthermore, they ignore the fact that the interpretation of histological lesions in biopsies is performed on serial sections. Serial sections help to avoid this type of error.. Marsh and his team did not demonstrate the SEM of a histological lesion that could be clearly classified as type IIIa or b in the conventional histology. In our opinion such an attempt would have lead to a dismissal of their proposal.

Furthermore, the authors were also prey to an error in their conception they suggest that an early lesion without villous flattening (type I or II lesion) turns into a flat mucosa without intermediary stages. Therefore, the villi would show a normal shape in the type I or II lesions, and then immediately become flat. We do not think that this is possible.
